# Supplementary material for: Association between oral microbiome and seven types of cancers in East Asian population: a two-sample Mendelian randomization analysis
Source: Front Mol Biosci. 2023 Nov 21;10:1327893. doi: 10.3389/fmolb.2023.1327893 (PMC10702768; doi:10.3389/fmolb.2023.1327893)
Supplement: Supplementary file 1 [file DataSheet1.ZIP › Supplementary Materials/MR plots for tongue/tongue═╝/Pancreatic cancer/╥╚╧┘░⌐_╔╕╤í╜ß╣√/pheno.486_to_pancreatic cancer_leave_one_out.pdf]

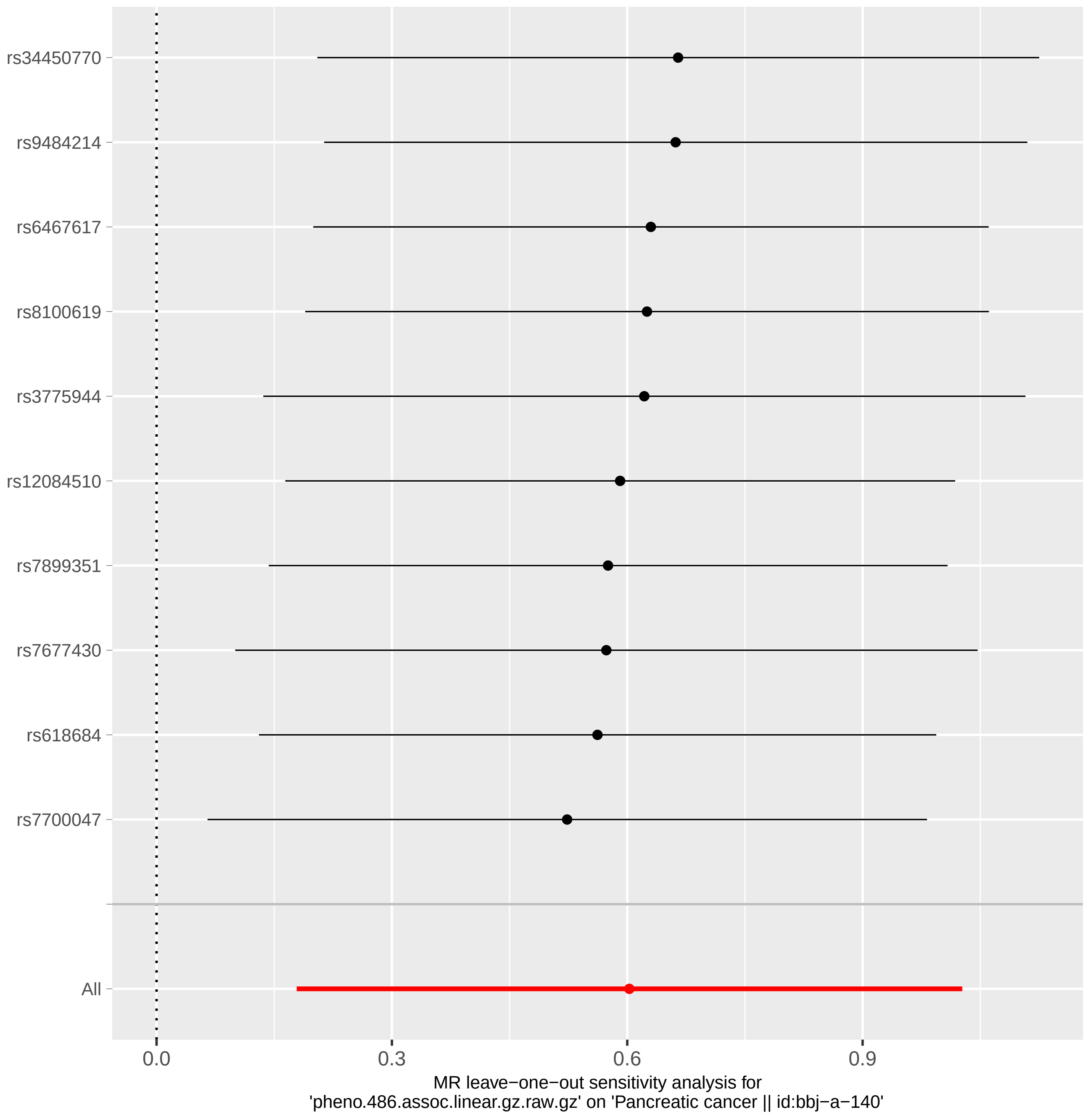
rs34450770
rs9484214
rs6467617
rs8100619
rs3775944
rs12084510
rs7899351
rs7677430
rs618684
rs7700047
All
0.0
0.3
0.6
0.9
MR leave−one−out sensitivity analysis for
'pheno.486.assoc.linear.gz.raw.gz' on 'Pancreatic cancer || id:bbj−a−140'
